# Hepatocyte growth factor as a driver of synovial inflammation and therapeutic resistance in rheumatoid arthritis

**DOI:** 10.3389/fimmu.2026.1718591

**Published:** 2026-01-30

**Authors:** Quang Minh Dang, Ryu Watanabe, Takeshi Iwasaki, Ryuhei Ishihara, Keiichiro Kadoba, Koichi Murata, Masao Tanaka, Hiromu Ito, Thanh Duc Tran, Sang Quang Tran Nguyen, Khanh Van Nguyen, Hongxin Sun, Masao Katsushima, Hui Zhang, Yutaro Yamada, Kenji Mamoto, Tadashi Okano, Tran Trung Dung, Le Thi Thanh Thuy, Motomu Hashimoto

**Affiliations:** 1 Department of Clinical Immunology, Osaka Metropolitan University Graduate School of Medicine, Osaka, Japan; 2 Department of Orthopaedic Surgery, College of Health Science, VinUniversity, Hanoi, Vietnam; 3 Sarcoma Center, Vinmec Healthcare System, Hanoi, Vietnam; 4 Department of Rheumatology and Clinical Immunology, Kyoto University Graduate School of Medicine, Kyoto, Japan; 5 Department of Orthopaedic Surgery, Graduate School of Medicine, Kyoto University, Kyoto, Japan; 6 Department of Advanced Medicine for Rheumatic Diseases, Graduate School of Medicine, Kyoto University, Kyoto, Japan; 7 Department of Orthopaedic Surgery, Kurashiki Central Hospital, Kurashiki, Japan; 8 Department of Orthopaedic Surgery, Osaka Metropolitan University Graduate School of Medicine, Osaka, Japan; 9 Department of Hepatology, Osaka Metropolitan University Graduate School of Medicine, Osaka, Japan; 10 Anatomic pathologist Pathology division of Laboratory Department, Vinmec Healthcare System, Hanoi, Vietnam; 11 Department of Rheumatology and Clinical Immunology, The First Affiliated Hospital of Sun Yat-sen University, Guangzhou, Guangdong, China; 12 Center for Senile Degenerative Disorders (CSDD), Osaka Metropolitan University Graduate School of Medicine, Osaka, Japan; 13 Department of Global Education and Medical Sciences, Osaka Metropolitan University Graduate School of Medicine, Osaka, Japan

**Keywords:** hepatocyte growth factor, interleukin-6 (IL-6), rheumatoid arthritis, synovial fibroblast, toll-like receptor

## Abstract

**Background:**

Previous studies have demonstrated that hepatocyte growth factor (HGF) is implicated in treatment resistance in rheumatoid arthritis (RA). This study aimed to elucidate the mechanistic role of HGF in synovial inflammation and assess the therapeutic potential of targeting the HGF-c-Met axis.

**Methods:**

Plasma HGF levels were measured in 66 RA patients. The expression of HGF and its receptor, c-Met, in synovial tissue was assessed using publicly available single-cell RNA sequencing data and immunostaining. The effects of HGF on synovial fibroblasts were examined through bulk RNA sequencing, immunostaining, and quantitative PCR. The therapeutic efficacy of the c-Met inhibitor savolitinib was evaluated in a mouse model of arthritis.

**Results:**

Plasma HGF levels were significantly elevated in RA patients (*p* = 0.0003) and correlated with Disease Activity Score 28-ESR (r = 0.367, *p* = 0.002). Single-cell analysis and immunostaining revealed that HGF was predominantly expressed in monocytes and fibroblasts, while c-Met expression was restricted to synovial fibroblasts. RNA sequencing indicated that HGF stimulation upregulated key inflammatory markers, including IL-6 and HGF itself, in synovial fibroblasts, establishing an inflammatory feedback loop. *In vivo*, inhibition of the HGF-c-Met pathway with savolitinib significantly suppressed arthritis development and reduced synovial inflammation. Additionally, activation of Toll-like receptor 4 and 5 activation induced HGF production in human monocytes, which may amplify IL-6-mediated inflammation.

**Conclusion:**

HGF primarily acts on synovial fibroblasts, driving an IL-6-mediated inflammatory amplification loop that may contribute to therapeutic resistance in RA. Targeting the HGF-c-Met pathway could represent a novel strategy for overcoming treatment refractoriness in RA.

## Introduction

Recent advancements in the treatment of rheumatoid arthritis (RA) have markedly improved patient outcomes. In particular, the broad implementation of a treat-to-target strategy, which often combines high-dose methotrexate with biological or targeted synthetic disease-modifying antirheumatic drugs (b/tsDMARDs), has enabled many patients to achieve remission at clinical, structural, and functional levels (1). Nevertheless, a subset of patients exhibits a refractory clinical course in which disease activity remains uncontrolled despite the use of two or more b/tsDMARDs with distinct mechanisms of action. Such cases are now classified as difficult-to-treat RA (D2T RA) (2). Previous studies have identified several risk factors associated with D2T RA, including elevated baseline disease activity, high rheumatoid factor titres, pulmonary involvement, delayed diagnosis, and postponed initiation of b/tsDMARD therapy (3–8).

Timely administration of the most appropriate therapeutic agent is essential to prevent progression to D2T RA. We have previously conducted multi-omics studies to predict drug efficacy in advance, thereby facilitating optimal drug selection. These studies integrated flow cytometric, transcriptomic, and plasma proteomic analyses of peripheral blood samples (9, 10). In patients refractory to anti-tumor necrosis factor (TNF) inhibitors, elevated levels of hepatocyte growth factor (HGF) and C-X-C motif chemokine ligand 10 (CXCL10) were observed prior to treatment initiation (10). Similarly, in patients who showed inadequate response to abatacept, increased levels of HGF and HGF-producing monocytes were detected in the peripheral blood at baseline (9). These findings suggest that HGF may be a shared biomarker linked to treatment resistance to these therapies.

HGF is a pleiotropic cytokine initially identified as a hepatocyte proliferation stimulant (11). Further research has revealed that HGF, through binding to its specific receptor c-Met, mediates diverse biological effects, including cell proliferation, survival, angiogenesis, tissue regeneration and wound healing (12, 13). Conversely, dysregulation of HGF/c-Met signaling has been implicated in various diseases, such as fibrotic disorders, cardiovascular diseases, cancer progression and metastasis (14, 15).

Elevated HGF levels in the blood and synovial tissues have been documented in RA patients (16–19). HGF is thought to contribute to RA pathogenesis by promoting monocyte migration and osteoclastogenesis in experimental arthritis model (20, 21); however, the exact mechanisms through which HGF impacts therapeutic outcomes in patients with RA remain unclear. This study aimed to elucidate the mechanistic role of HGF in the pathophysiology of RA and treatment resistance.

## Methods

### Study design and clinical evaluation of patients

Patients who visited Kyoto University Hospital between April 2020 and March 2021 and had been treated with a treat-to-target strategy (22) were cross-sectionally enrolled. The diagnosis of RA was based on the 1987 or 2010 classification criteria (23, 24). The medical records of the enrolled patients were retrospectively reviewed. Disease activity of RA was evaluated using the Disease Activity Score 28-erythrocyte sedimentation rate (DAS28-ESR), DAS28-C-reactive protein (DAS28-CRP), simplified disease activity index (SDAI), and clinical disease activity index (CDAI). Age- and sex-matched individuals who were referred to Osaka Metropolitan University between May 2021 and March 2022 on suspicion of autoimmune diseases but were not diagnosed with any disease were considered healthy controls.

### Measurement of plasma protein levels

Plasma protein levels of interleukin (IL)-1β, IL-2, IL-4, IL-6, IL-10, IL-17, interferon (IFN)-α, IFN-β, IFN-γ, tumor necrosis factor (TNF)-α, dickkopf-1 (Dkk-1), C-C motif chemokine ligand 2 (CCL2), CXCL10, and HGF were evaluated using the Luminex^®^ Discovery Assay Human Premixed Multi-Analyte Kit (Cat No. LXSAHM-20; R&D Systems Inc., Minneapolis, MN, USA) according to the protocol provided by the manufacturer, as previously described (25).

### Human synovial tissue samples

We obtained synovial tissue samples during the surgical procedure from 16 patients with RA and 4 patients with osteoarthritis (OA) undergoing orthopedic surgery at the Department of Orthopaedic Surgery, Osaka Metropolitan University Hospital.

### Isolation and cell culture of fibroblasts from human synovial tissue

Synovial tissue samples from RA patients were finely minced, digested for 30 min at 37 °C in Hank’s Balanced Salt Solution (#084-08965, FUJIFILM, Osaka, Japan), containing collagenase D (#11088858001, Merck, Germany), and thereafter digested for 15 min at 37 °C in DNase buffer containing DNase I (#314-08071, Nippon Gene, Japan). The cell suspension was then filtered and the cells were collected by centrifugation at 1800 rpm for 5 min. Cells were kept in primary culture for 7 days in complete medium consisting of low glucose DMEM/10% fetal calf serum (FCS), 1% Penicillin/Streptomycin, including removal of non-adherent cells on days 1, 3, 5, and 7 and incubated at 37 °C, 5% CO2 (26, 27). We confirmed that over 97% of these cells were positive for the fibroblast marker CD90 (28). Synovial fibroblasts were seeded in 6-well plates (BD Biosciences, Bedford, MA, USA) at a density of 1 × 10^5^ cells per well and maintained in complete medium.

### Library preparation and RNA sequencing

Total RNA (>50 ng) obtained from each synovial fibroblast sample was subjected to a sequencing library construction using TruSeq Stranded mRNA Library Prep Kit (Illumina, Inc., San Diego, CA) according to the manufacturer’s protocols. The equally pooled libraries of the samples were sequenced using NovaSeq 6000 (Illumina, Inc.) in 101-base-pair (bp) paired-end reads. Sequencing reads were trimmed using Trimmomatic ver. 0.36 (29) (leading: 20, trailing: 20, slidingwindow: 4:15, minlen: 36) and aligned to hg38 reference genome using STAR (ver. 2.7.3a) (30). Gene counts were generated by RSEM (ver. 1.3.1) (31) using Homo_sapiens.GRCh38.95.gtf from the Ensembl database (32). Gene counts were normalized by size factor implemented in DESeq2 (33). Differential gene expression test was performed by the Wald test using DESeq2. Overrepresentation analysis of gene sets was performed using Metascape (34) with default settings.

### Mice

SKG mice (35) were obtained from CLEA Japan, as previously described (36). Eight-week-old female SKG mice were maintained under specific pathogen-free conditions at a constant temperature (23 ± 1°C) with a 12-hour light/dark cycle. Arthritis was induced by a single intraperitoneal injection of 2 mg of Zymosan (Z4250, Merck, Japan) (37). Joint swelling was assessed by visual inspection, and arthritis scores were evaluated as previously described (37). Treatment with savolitinib (AZD6094, Selleck, Japan) was initiated on day 7 at a dose of 2.5 mg/kg/day (20), dissolved in saline, and administered orally once daily for four weeks. Control mice received daily saline.

### Statistical analysis

Statistical analyses were performed using GraphPad Prism 10 (GraphPad Software Inc., La Jolla, CA, USA). The normality of all data was evaluated using Kolmogorov–Smirnov tests. Statistical significance was determined using unpaired two-tailed Student *t*-tests for normally distributed data, and Mann–Whitney U tests for data that were not normally distributed. One-way or two-way ANOVA with Tukey’s multiple comparison test was used for 3 or 4 group comparison. Categorical variables were analyzed using Fisher’s exact test. Correlation was assessed using Spearman analysis. Values with p < 0.05 were considered significant.

### Supplementary methods

Additional methodological details are provided online.

## Results

### Comparison of plasma protein levels between patients with RA and healthy controls

**Figure 1:**
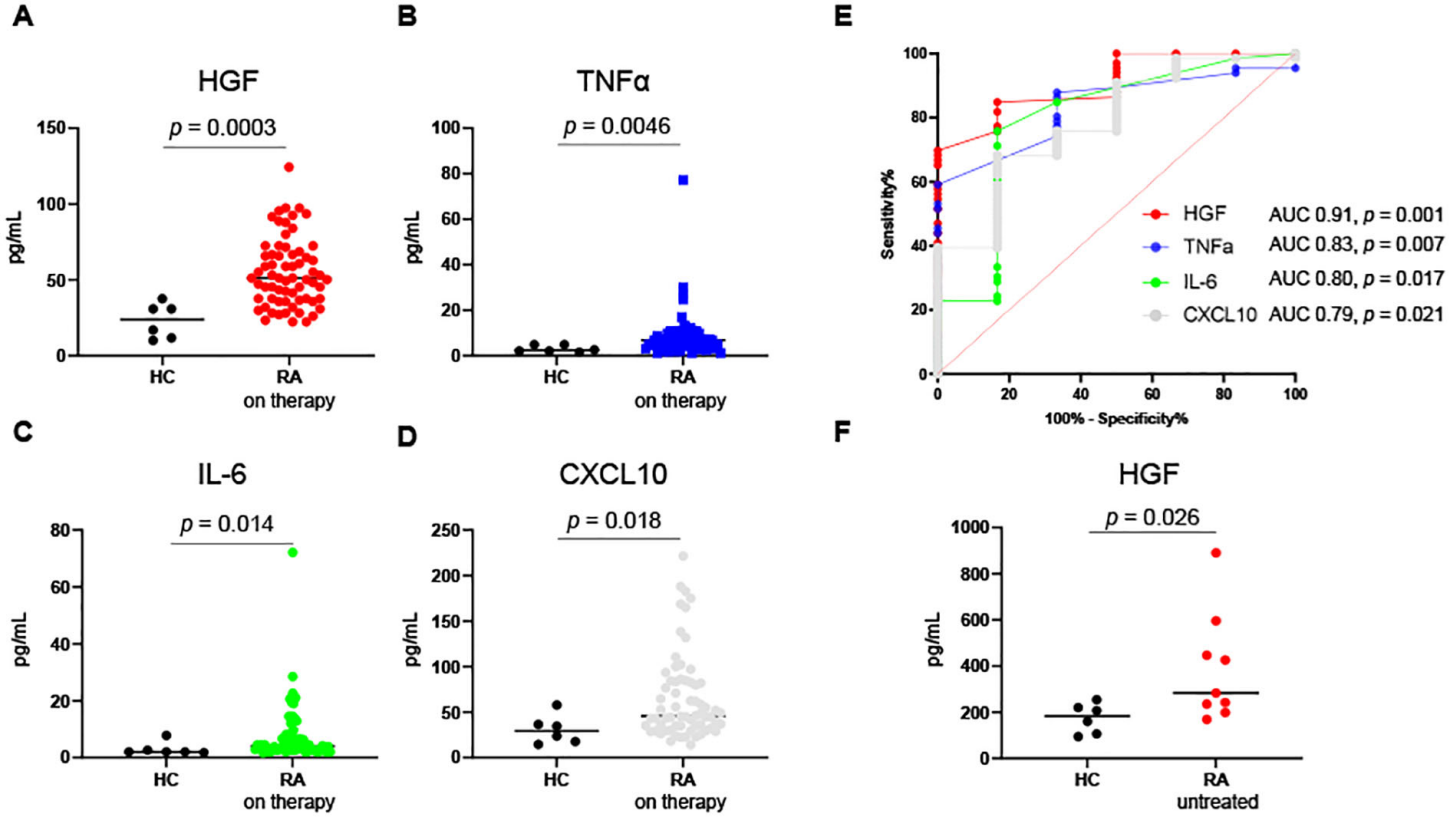
Comparison of plasma concentrations of HGF, TNFα, IL-6, and CXCL10 between patients with RA and healthy controls. Plasma concentrations of HGF **(A)**, TNFα **(B)**, IL-6 **(C)** and CXCL10 **(D)** were compared between 66 patients with RA on therapy and 6 healthy controls (HC). All data are median with individual values. Mann-Whitney U test. **(E)** Receiver operating characteristic analysis of these four proteins to determine the specificity and sensitivity for distinguishing between RA and HC. **(F)** Serum concentrations of HGF were compared between 9 patients with untreated RA and 6 HC. Mann-Whitney U test. AUC, area under the curve; CXCL10, C-X-C motif chemokine ligand 10; HGF, hepatocyte growth factor; IL-6, interleukin-6; RA: rheumatoid arthritis; TNFα, tumor necrosis factor α.

Our previous studies have demonstrated that HGF predicts resistance to treatment with anti-TNF inhibitors and abatacept prior to treatment initiation. Specifically, plasma concentrations of HGF were significantly higher in non-responders compared to responders to these therapies at baseline (9, 10). To further investigate this finding, we analyzed 66 blood samples from an independent cross-sectional cohort to assess whether HGF levels remains elevated in patients undergoing therapy. The demographic characteristics of the patients are summarized in **Supplementary Table 1**. The median DAS28-ESR was 2.7, indicating that disease activity in these patients were relatively controlled. Among the 14 plasma proteins measured (**Supplementary Table 2**), plasma concentrations of IL-6, TNF-α, CXCL10, and HGF were significantly higher in patients with RA compared to healthy controls (*p* = 0.014, *p* = 0.0046, *p* = 0.018, and *p* = 0.0003, respectively). Notably, HGF levels remained significantly elevated even after adjustment for multiple comparisons using the Bonferroni correction (significance threshold: *p* = 0.0036). **Figure 1** provides a visual comparison of plasma concentrations of IL-6, TNF-a, CXCL10, and HGF (**Figures 1A–D**). Receiver operating characteristic (ROC) analysis revealed that among these four proteins, HGF exhibited the highest specificity and sensitivity for distinguishing between healthy controls and RA patients (area under the curve 0.91; 95% confidence interval [CI] 0.81–1.0, *p* = 0.001; **Figure 1E**).

In addition, in a separate cohort, we compared serum HGF levels in untreated RA patients with those in age- and sex-matched healthy controls using the same assay employed for plasma proteins. HGF levels were also significantly elevated in untreated RA patients (*p* = 0.026, **Figure 1F**). Together, these findings suggest that HGF concentrations remain persistently elevated not only in untreated RA patients but also in patients receiving apparently effective therapy.

### Correlation between plasma HGF concentrations and disease activity in RA

**Figure 2:**
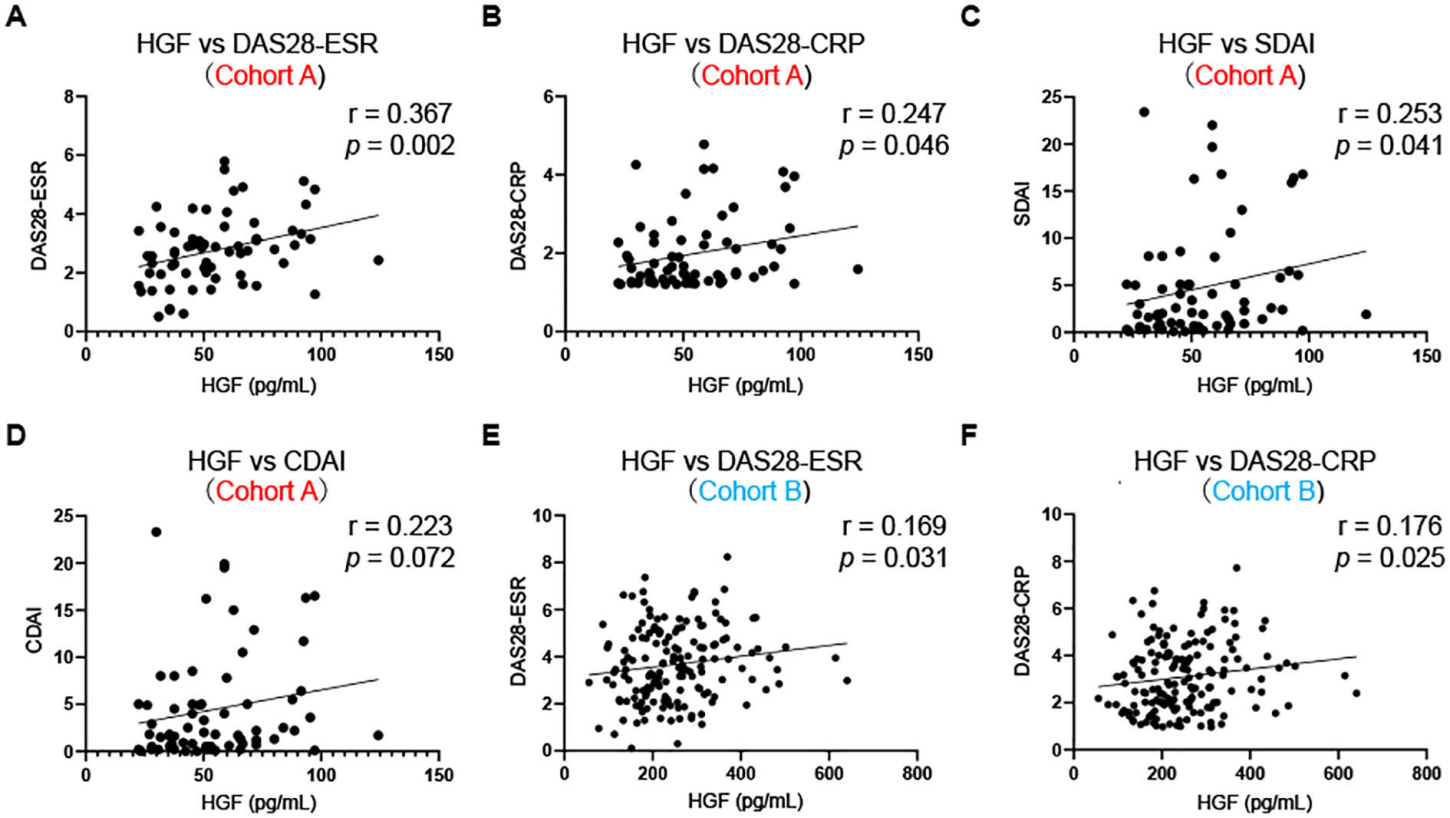
Correlation between plasma HGF concentrations and disease activity in RA. Correlation between plasma HGF concentrations and DAS28-ESR **(A)**, DAS28-CRP **(B)**, SDAI **(C)**, and CDAI **(D)** in cohort A, and **(E)** DAS28-ESR and **(F)** DAS28-CRP in cohort **(B)** Cohort A includes 66 RA patients undergoing therapy, while cohort B includes 164 RA patients prior to the initiation of biologic agents. Spearman correlation analyses. CDAI, clinical disease activity index; DAS28-CRP, Disease Activity Score 28- C-reactive protein; DAS28-ESR, DAS28-erythrocyte sedimentation rate; HGF, hepatocyte growth factor; RA: rheumatoid arthritis; SDAI, simplified disease activity index.

We then investigated the correlation between plasma HGF levels and disease activity in 66 patients with RA (**Figures 2A–D**, Cohort A). Plasma HGF levels were significantly associated with DAS28-ESR (r = 0.37, 95% CI: 0.13 to 0.56, *p* = 0.002), DAS28-CRP (r = 0.25, 95% CI: -0.0022 to 0.47, *p* = 0.046), and SDAI (r = 0.25, 95% CI: 0.0041 to 0.47, *p* = 0.041), but not CDAI (r = 0.22, 95% CI: -0.028 to 0.45, *p* = 0.072). To validate these findings, we analyzed 164 blood samples collected prior to the initiation of biologic agents in the previous studies (Cohort B) (9, 10). Consistent with the initial analysis, plasma HGF levels were significantly correlated with DAS28-ESR (r = 0.17, 95% CI: 0.011 to 0.32, *p* = 0.031) and DAS28-CRP (r = 0.18, 95% CI: 0.018 to 0.32, *p* = 0.025). These findings suggest that plasma HGF concentrations are positively correlated with disease activity in RA, even during treatment.

### Expression of HGF and c-met in RA synovial tissue

**Figure 3:**
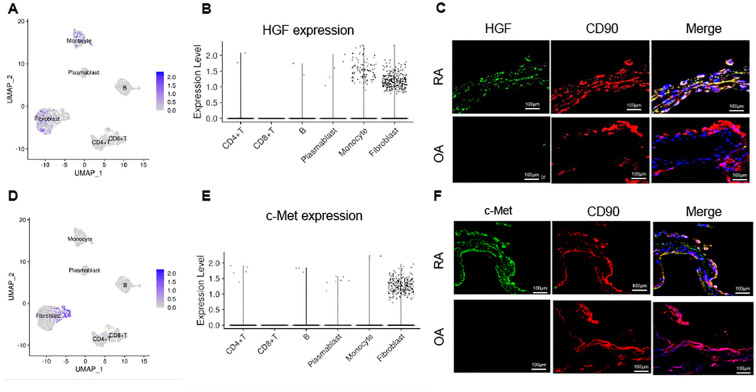
Expression of HGF and c-met in RA synovial tissue. **(A, B, D, E)** Single-cell RNA sequencing data from the AMP project (38) were analyzed to determine HGF and c-Met expression in RA synovial tissue. UMAP plots (A for HGF and D for c-Met) and violin plots (B for HGF and E for c-Met) are shown. **(C, F)** Immunohistochemical staining for HGF and c-Met using synovial tissue from OA and RA. Representative images from 3 independent samples were shown. Anti-HGF antibody (green), anti-c-Met antibody (green), anti- CD90 antibody (red), and DAPI (blue) were used. Scale bar indicates 100 μm. HGF, hepatocyte growth factor; OA, osteoarthritis; RA, rheumatoid arthritis.

To assess the expression of HGF in RA synovial tissue, we analyzed single-cell RNA sequencing data from the Accelerating Medicines Partnership (AMP) project (38). The analysis revealed that HGF expression was predominantly localized to monocytes and fibroblasts (**Figures 3A, B**). Immunohistochemical staining demonstrated robust HGF expression in RA synovial fibroblasts compared to those in OA synovial tissue (**Figure 3C**). Similarly, single-cell RNA sequencing data indicated that c-Met, the receptor for HGF, was almost exclusively expressed in fibroblasts (**Figures 3D, E**). Immunohistological analysis further confirmed that RA synovial fibroblasts exhibited higher c-Met expression compared to OA synovial fibroblasts (**Figure 3F**). These findings indicate that monocytes and synovial fibroblasts are the primary sources of HGF production, which may affect synovial fibroblasts via c-Met in RA synovial tissue.

### HGF biases synovial fibroblasts toward an inflammatory phenotype

**Figure 4:**
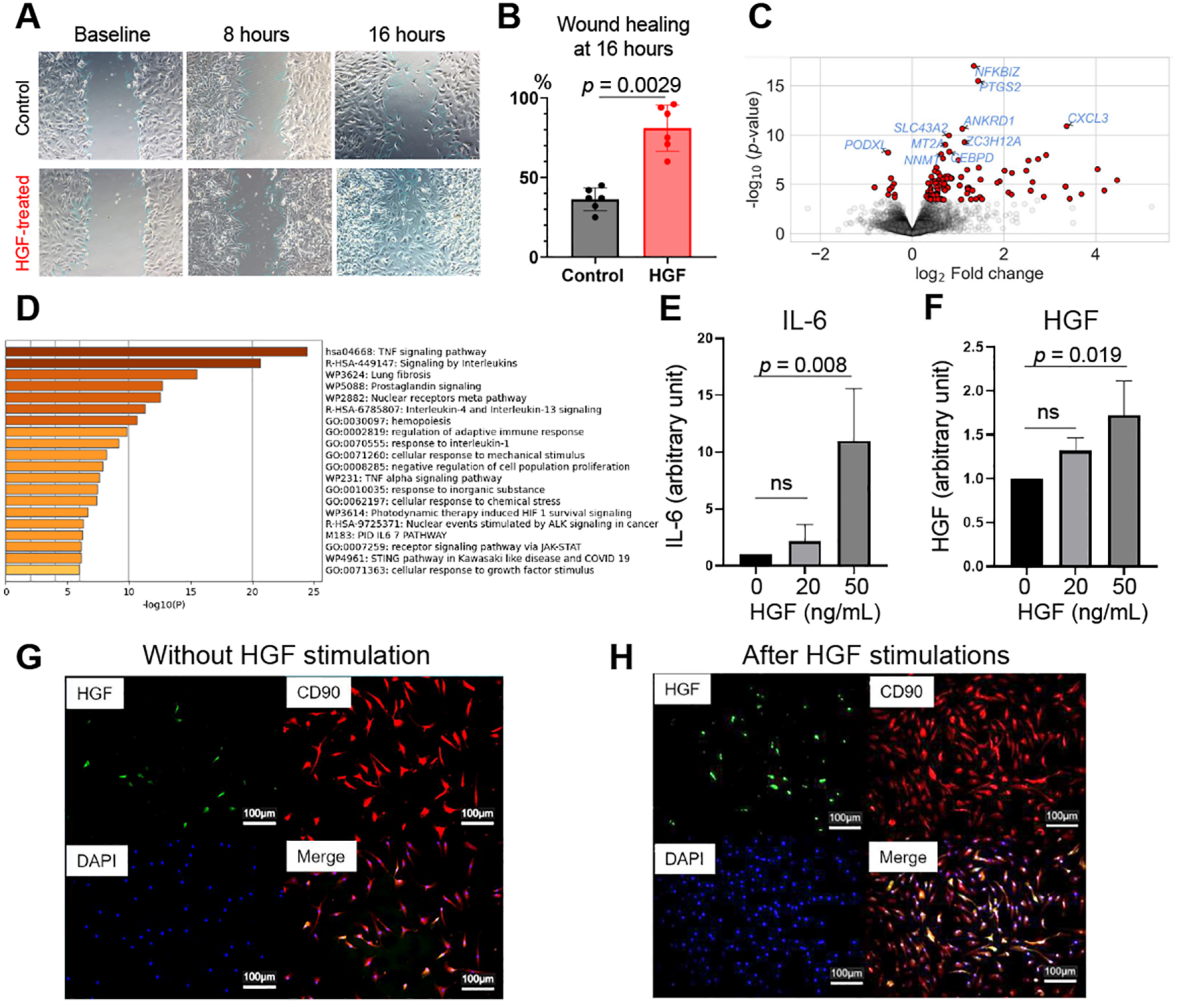
HGF stimulates synovial fibroblasts to produce IL-6 and HGF itself. **(A, B)** A uniform, cell-free wound area was generated in confluent synovial fibroblasts from RA patients by scratching with a sterile pipette tip. The cells were then incubated in DMEM containing 10% FCS, supplemented with either the vehicle control (DMSO) or HGF (50 ng/mL). Cell migration was quantified by measuring the width of the wound in each well. The wound area at 0 hours was defined as 100% open, and the percentage of wound closure at subsequent time points was calculated relative to this baseline. Detailed methods are described in Supplementary Methods. Data represent mean ± SD of 6 samples. Paired *t* test. **(C, D)** Synovial fibroblasts from three RA patients were stimulated with or without HGF (50 ng/mL) for 6 hours and differentially expressed genes were illustrated as a volcano plot **(C)**. Each dot represents an individual gene, with red dots indicating genes significantly downregulated or upregulated (false discovery rate <0.05). An increased x-axis value indicates increased expression following HGF treatment. **(D)** Enrichment analysis of the 105 differentially expressed genes following HGF treatment. **(E, F)** Synovial fibroblasts from RA were stimulated with HGF at indicated concentrations for 6 hours and the expression of IL-6 **(E)** and HGF **(F)** were quantified by qPCR. Data represent mean ± SD of 3 samples. One-way ANOVA with Tukey’s multiple comparison test was used. **(G, H)** Synovial fibroblasts from RA were stimulated with or without HGF (50 ng/mL) for 6 hours and immunohistochemical staining for HGF was performed. Anti-HGF antibody (green), anti-CD90 antibody (red), and DAPI (blue) were used. Scale bar indicates 100 μm. Representative images from 3 independent experiments were shown. ns, not significant; HGF, hepatocyte growth factor; RA, rheumatoid arthritis.

To elucidate the mechanistic role of HGF on synovial fibroblasts, we first performed a wound healing assay to evaluate whether HGF promotes fibroblast proliferation and migration (39). Compared with control synovial fibroblasts, cells treated with 50 ng/mL HGF exhibited accelerated proliferation and migration, and HGF significantly enhanced wound healing at 16 hours (**Figures 4A, B**).

Next, we performed bulk RNA sequencing on synovial fibroblasts derived from three RA patients with or without HGF stimulation (i.e. in total, six specimens), and compared HGF-stimulated specimens with non-stimulated specimens. We found that 105 genes were differentially expressed following HGF treatment (**Figure 4C**; **Supplementary Table 3**). The gene set enrichment analysis of the 105 genes using Metascape revealed significant upregulation of pathways associated with TNF, IL-1, and IL-6 signaling (**Figure 4D**).

To further clarify the downstream effects of HGF on gene regulation, we mapped differential gene expression test results onto the most strongly dysregulated pathway—the TNF signaling pathway—using Pathview (40). This analysis revealed that several genes within the MAPK pathway were upregulated, with IL-1 and IL-6 positioned downstream of these alterations (**Supplementary Figure 1**). These findings provide additional insight into how HGF-induced perturbation of the TNF signaling pathway may influence downstream inflammatory responses.

Given that fibroblasts are a major source of IL-6 in the RA synovium (38), we quantified IL-6 production following HGF stimulation using qPCR. The results demonstrated that HGF stimulated IL-6 production in a dose-dependent manner in synovial fibroblasts (**Figure 4E**). Furthermore, HGF treatment upregulated the expression of HGF itself in a dose-dependent manner (**Figure 4F**). Immunohistochemical analysis corroborated these findings, revealing that HGF promotes its own production in synovial fibroblasts (**Figures 4G, H**). These findings suggest the presence of an inflammatory feedback loop mediated by HGF, amplifying IL-6-medicated synovial inflammation.

### Targeting the HGF-c-Met axis suppresses arthritis development

**Figure 5:**
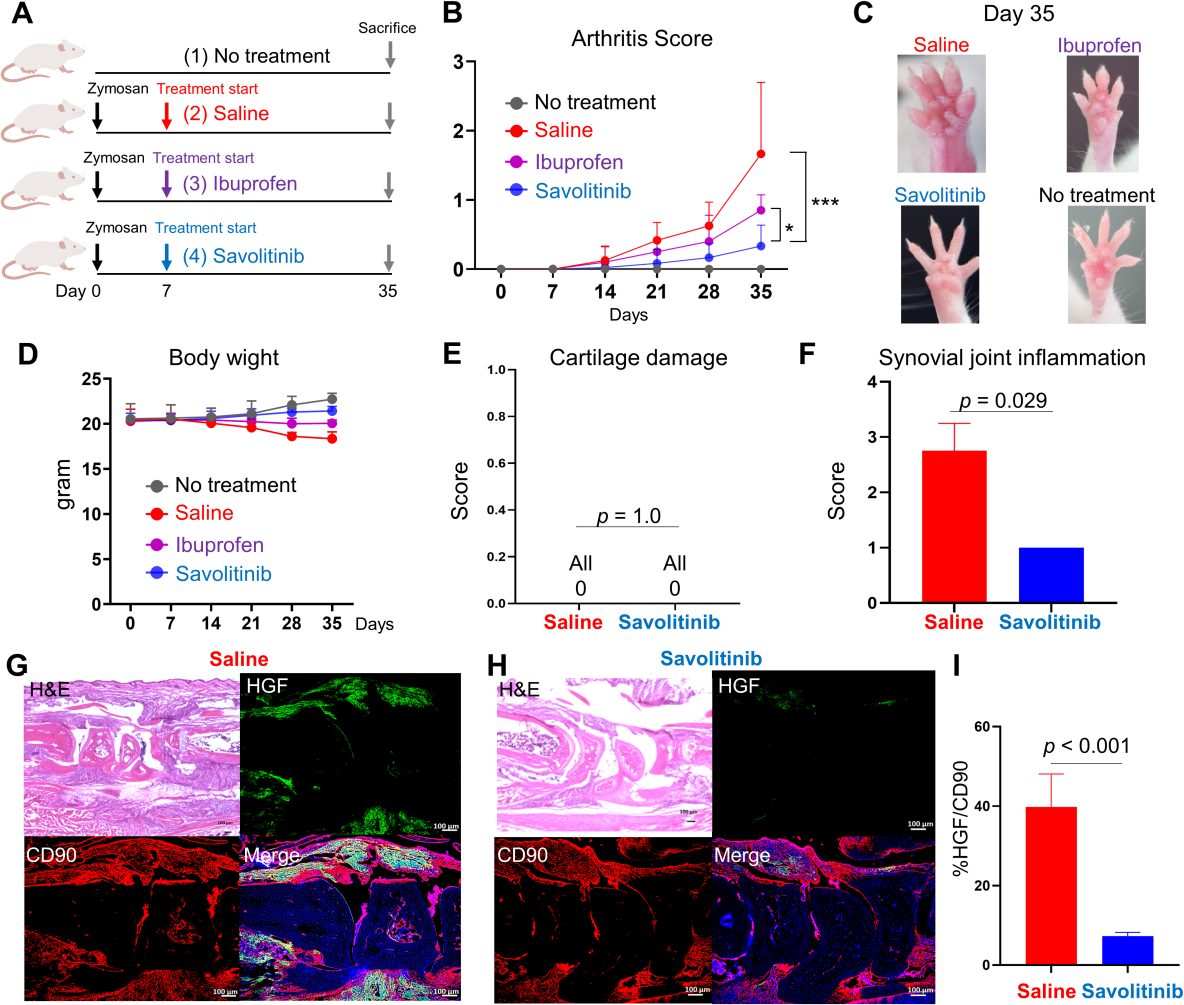
Targeting the HGF-c-Met axis suppresses arthritis development. **(A)** Scheme of animal experiments. Arthritis was induced by a single intraperitoneal injection of 2 mg of Zymosan, and arthritis scores were evaluated until day 35. Treatment with savolitinib (n = 6) was initiated on day 7 at a dose of 2.5 mg/kg/day, dissolved in saline, and administered orally once daily for four weeks. As controls, we included (1) a group without zymosan injection (n = 5), (2) a group that received daily saline after zymosan injection (n = 6), and (3) a group treated with ibuprofen (30 mg/kg/day) following zymosan injection (n = 5). **(B)** Arthritis scores (mean ± SD) were evaluated in each treatment group until day 35. **(C)** Representative images of mouse forepaws from each experimental group on day 35. **(D)** Changes in body weight (mean ± SD) during the treatment period. **(E, F)** Cartilage damage scores **(E)** and synovial inflammation scores **(F)** were assessed in the saline-treated group and the savolitinib-treated group. Mann-Whitney U test. **(G, H)** Hematoxylin and Eosin (H&E) staining images and immunofluorescence staining images of wrist joints in the saline group **(G)** and in the savolitinib group **(H)**. Representative images are shown. Anti-HGF antibody (green), anti-CD90 antibody (red), and DAPI (blue) were used. Scale bar indicates 100 μm. **(I)** Percentage of HGF-positive cells among CD90-positive cells in the saline group and in the savolitinib group. Data represent mean ± SD. ***p < 0.001, *p < 0.05; HGF, hepatocyte growth factor; ns, not significant.

To further elucidate the role of the HGF-c-Met pathway in arthritis, we examined whether savolitinib, a selective c-Met inhibitor, could suppress arthritis onset in a mouse model. SKG mice received oral administration of savolitinib at a dose of 2.5 mg/kg/day for four weeks, starting one week after Zymosan injection. As controls, we included [i] a group without zymosan injection, [ii] a group that received daily saline after zymosan injection, and [iii] a group treated with ibuprofen (30 mg/kg/day) following zymosan injection. Arthritis scores were assessed until day 35 (**Figure 5A**). Savolitinib treatment significantly suppressed arthritis development compared with both saline-treated and ibuprofen-treated mice (**Figures 5B, C**).

To assess treatment safety, we monitored body weight throughout the experiment and observed no weight loss or other overt signs of toxicity in savolitinib-treated mice (**Figure 5D**). Histological evaluation of the liver and kidneys further confirmed the absence of apparent drug-induced abnormalities (**Supplementary Figure 2**).

We next evaluated the extent of joint structural damage in saline-treated and savolitinib-treated mice based on the scoring methodology described by Hayer et al. (41). Cartilage damage scores were zero in both saline-treated and savolitinib-treated groups, with no significant difference between them (**Figure 5E**). In contrast, scores for synovial joint inflammation were significantly reduced in the savolitinib-treated group (**Figure 5F**).

We further examined HGF expression by immunohistochemistry. As shown in **Supplementary Figure 3**, mice that did not receive zymosan and did not develop arthritis exhibited minimal HGF expression. In saline-treated arthritic mice, however, HGF was strongly expressed at inflamed joints, whereas savolitinib treatment markedly reduced HGF expression (**Figures 5G, H**). In addition, the proportion of HGF-positive cells among CD90-positive fibroblasts was significantly decreased following savolitinib treatment (**Figure 5I**). These results suggest that targeting the HGF–c-Met axis with savolitinib effectively suppresses arthritis development by reducing synovial inflammation, while demonstrating no detectable toxicity.

### HGF production by monocytes upon stimulation of Toll-like receptors

**Figure 6:**
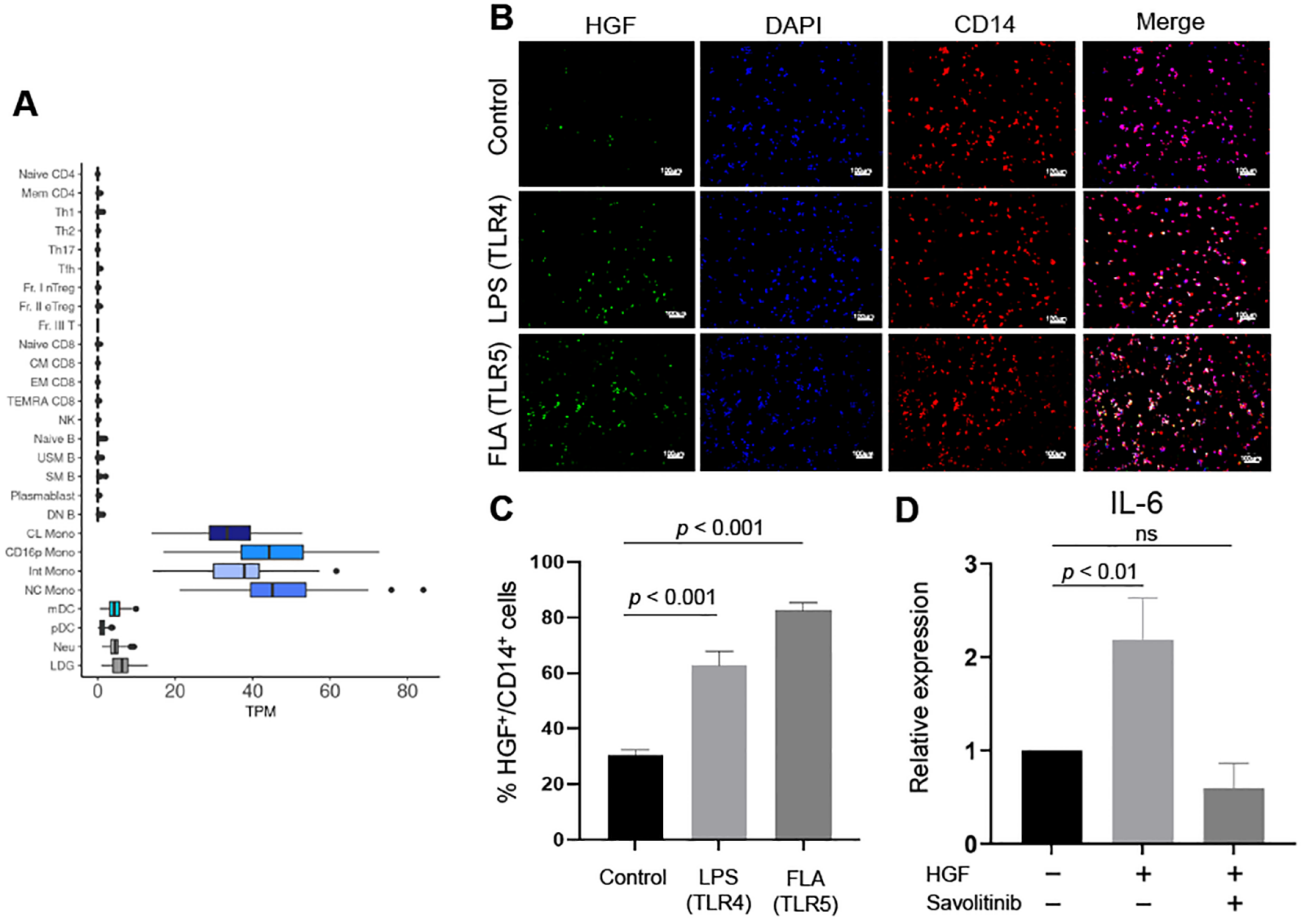
HGF production by monocytes upon stimulation of Toll-like receptors (TLRs). **(A)** Expression of HGF in each cell type in healthy individuals. HGF was primarily expressed by classical (CL), CD16-positive (CD16p), intermediate (Int), and non-classical (NC) monocytes. **(B, C)** Human CD14^+^ monocytes were freshly isolated and stimulated with Toll-like receptor (TLR) stimulants. LPS stimulates TLR4, while FLA stimulates TLR5. After 6 hours, immunohistochemical staining for HGF was performed. Anti-HGF antibody (green), anti- CD14 antibody (red), and DAPI (blue) were used. Scale bar indicates 100 μm. Representative images from 3 independent experiments were shown. **(C)** Percentage of HGF+ cells out of CD14+ monocytes was shown. Data represent mean ± SD of 3 independent experiments. One-way ANOVA with Tukey’s multiple comparison test was used. **(D)** Human CD14+ monocytes from healthy controls were stimulated 1) without HGF, 2) with HGF (50 ng/mL), or 3) with HGF (50 ng/mL) and savolitinib (10 μM) for 6 hours and the expression of IL-6 was quantified by qPCR. Data represent mean ± SD of 3 samples. One-way ANOVA with Tukey’s multiple comparison test was used. HGF, hepatocyte growth factor; LPS, lipopolysaccharide; FLA, flagellin.

To investigate the upstream events driving the HGF-mediated inflammatory loop, we examined the role of Toll-like receptor (TLR) stimulation. This focus was based on our previous studies, which demonstrated that HGF is predominantly produced by monocytes with high expression of TLR5 in peripheral blood from RA patients (9). Supporting this observation, bulk RNA sequencing data of each immune cell subsets in healthy individuals (https://www.immunexut.org/) (42) confirmed that HGF production is primarily attributed to monocytes (**Figure 6A**). Immunohistochemical staining further revealed that stimulation of not only TLR5 but also TLR4 significantly enhanced HGF production in human peripheral blood monocytes (**Figures 6B, C**). These findings suggest that TLR-stimulated monocytes are a major source of HGF, which may initiate and sustain the HGF-IL-6-mediated amplification of inflammatory responses.

To further examine the functional consequences of HGF signaling in monocytes, we assessed whether HGF exerts autocrine effects on these cells. Human peripheral blood monocytes were isolated and stimulated with recombinant HGF (50 ng/mL). HGF treatment significantly increased IL-6 production, indicating that HGF can directly promote pro-inflammatory activation of monocytes. Importantly, this HGF-induced IL-6 production was suppressed by savolitinib (**Figure 6D**). Although transcriptomic analysis of RA synovial tissue showed that expression of the HGF receptor c-Met is largely restricted to synovial fibroblasts (**Figure 3E**), our findings demonstrate that circulating monocytes are also responsive to HGF stimulation. These results suggest that HGF may contribute to RA pathogenesis not only through synovial fibroblast activation but also by enhancing IL-6 production from monocytes, thereby amplifying systemic and synovial inflammation.

## Discussion

This study provides compelling evidence that HGF plays a pivotal role in the inflammatory pathophysiology of RA and may contribute to therapeutic resistance. Elevated plasma HGF levels in RA patients, along with its robust expression in synovial tissues, particularly by monocytes and fibroblasts, suggest that HGF is not merely a biomarker but a functional mediator of this disease. These findings underscore the importance of the HGF-c-Met axis as a potential therapeutic target for treating RA and overcoming treatment refractoriness in RA.

Previous studies have demonstrated that HGF levels in the blood and synovial tissues are increased in RA patients compared to healthy controls and OA patients (16–19). Although effective treatment with biologics have been reported to decrease HGF levels in the blood (20), plasma HGF concentrations remain elevated even during treatment in our study (**Figure 1A**). In addition, plasma HGF levels were significantly associated with disease activity of RA prior to biologic therapy and even during treatment (**Figure 2**). These observations raise the possibility that current therapeutic regimens fail to fully suppress HGF-driven pathways, which may contribute to subclinical inflammation and the risk of disease flares. Our findings are consistent with prior report linking elevated HGF levels to poor treatment responses to anti-TNF therapy in Crohn’s disease (43).

Notably, our findings highlight the role of synovial fibroblasts as both a target and a mediator of HGF’s effects (**Figure 3**). These cells have emerged as key drivers of RA, contributing to joint destruction through their invasive phenotype and pro-inflammatory cytokine production (44–46). Indeed, it has been reported that baseline plasma HGF concentrations predict joint damage in RA (47), which is consistent with our results. The preferential expression of c-Met in fibroblasts suggests that these cells are primed to respond to HGF, further amplifying localized inflammation and potentially leading to resistance to conventional and targeted therapies. Recent studies have demonstrated that fibroblast activation correlates with refractory disease states, supporting the notion that targeting fibroblast-specific pathways may offer therapeutic benefits in D2T RA (48).

HGF plays critical physiological roles in diverse biological processes, including organogenesis, tissue regeneration, and cellular homeostasis. Its pleiotropic effects are mediated through binding to its receptor, c-Met, which activates a tyrosine kinase signaling cascade (49). Upon HGF binding, c-Met undergoes receptor dimerization and autophosphorylation of tyrosine residues within its intracellular domain. This phosphorylation event triggers the activation of multiple downstream signaling pathways, including the Ras/MAPK, PI3K/Akt, and STAT3 pathways (49). Consistent with these reports, our bulk RNA sequencing analysis revealed the upregulation of multiple signaling pathways in synovial fibroblasts following HGF stimulation (**Figures 4C, D**). Notably, upregulation of the TNF–MAPK signaling cascade suggested a potential mechanism by which HGF may drive downstream cytokine induction, including IL-1 and IL-6 (**Supplementary Figure 1**). Furthermore, HGF treatment not only enhanced IL-6 production but also upregulated its own expression, thereby establishing a self-perpetuating inflammatory feedback loop (**Figures 4F-H**). IL-6 is well-established as a central mediator in the pathogenesis of RA, exerting its effects by acting on synovial fibroblasts and promoting osteoclast differentiation through the upregulation of receptor activator of NF-κB ligand (RANKL) expression (50, 51). Therefore, this positive feedback loop involving HGF and IL-6 likely exacerbates synovial inflammation, amplifying the disease process.

Using a mouse model, we demonstrated that inhibition of the HGF-c-Met pathway effectively suppressed arthritis development (**Figure 5**). Savolitinib, a c-Met inhibitor, not only attenuated synovial inflammation but also reduced HGF production in the joints. Hosonuma et al. also investigated the effects of HGF-c-Met signal inhibition using savolitinib in SKG mice (20). While arthritis scores were comparable between control mice receiving saline and those treated with savolitinib, calcaneus bone volume loss was significantly suppressed in the savolitinib-treated group compared to the control group (20). This discrepancy in arthritis scores may be attributed to differences in the timing of treatment initiation. In our study, savolitinib administration began one week after Zymosan injection, whereas in their study, treatment was initiated two weeks post-injection. A delayed intervention may have allowed arthritis to progress to a more advanced stage, thereby diminishing the efficacy of c-Met inhibition. These findings suggest that early inhibition of the HGF-c-Met pathway may represent a novel therapeutic strategy for preventing RA progression.

Our previous studies demonstrated that HGF is predominantly produced by monocytes with high expression of TLR5 in peripheral blood from patients with RA (9). Consistent with these findings, the current study revealed that activation of TLR4 and TLR5 induces HGF production in CD14+ monocytes (**Figure 6**). The increased expression of HGF in response to TLR stimulation in monocytes suggests that innate immune activation acts as an upstream driver of inflammatory amplification. Moreover, TLR-mediated HGF production may serve as a critical link between systemic inflammation and localized synovial pathology in RA. In addition, previous studies have shown that IL-1β and TNFα stimulate HGF production in synovial fibroblasts (17, 20). The ability of diverse stimuli to induce HGF production in synovial fibroblasts may explain the persistently elevated levels of HGF observed in RA patients, even during treatment.

Our findings suggest that therapeutic inhibition of the HGF-c-Met axis could disrupt the inflammatory loop and mitigate treatment resistance in RA. Recently, inhibition of the HGF–c-Met pathway using PF-04217903 was reported to be effective in a model of gouty arthritis, where blockade of HGF–MET signaling promoted the resolution of neutrophilic inflammation by enhancing neutrophil apoptosis through caspase-3 cleavage (52). These findings further support the concept that pharmacological targeting of the HGF–c-Met axis can modulate inflammatory processes in diverse forms of arthritis.

Preclinical studies investigating the HGF-c-Met pathway in cancer and fibrotic diseases have demonstrated promising outcomes, including reduced fibrosis and suppression of tumor progression (53–57). A number of small-molecule inhibitors targeting the HGF–c-Met pathway have been developed, including altiratinib, bozitinib, capmatinib, and tivantinib, among others. These agents differ in their mechanisms of action, spectrum of kinase inhibition, and intended clinical indications, such as gastric and lung cancer (58). In the present study, we selected savolitinib because previous work had already examined its efficacy in SKG mice (20). Nevertheless, future studies employing alternative HGF–c-Met inhibitors will be important to determine whether the therapeutic effects observed here are specific to savolitinib or are a class effect. A targeted approach that selectively blocks HGF signaling in the synovium, while preserving its beneficial roles in tissue repair and regeneration could offer significant benefits in preventing the progression to D2T RA and improving outcomes for refractory RA.

The present study has several limitations. First, the study cohort consisted of a relatively small number of RA patients, and plasma HGF concentrations were not assessed longitudinally. Future research involving larger, longitudinal cohorts is needed to validate these findings and enhance their generalizability. Second, while previous studies have reported that HGF affects osteoclasts and neutrophils (21, 52), this study primarily focused on synovial fibroblasts and monocytes, and the effects of HGF on other cell types were not investigated. Third, despite the heterogeneity of synovial fibroblasts (45), we did not investigate which specific subpopulations contribute most significantly to HGF production. Further studies are warranted to elucidate the role of distinct fibroblast subsets in terms of HGF production. Fourth, our findings demonstrated that c-Met inhibitors effectively suppressed arthritis development. Additionally, Hosonuma et al. reported that savolitinib significantly reduced joint damage in established arthritis (20). However, the efficacy of c-Met inhibitors in treatment-resistant arthritis remains unclear. Finally, the clinical efficacy of c-Met inhibitors in RA patients has yet to be established and requires further investigation.

Despite these limitations, this study identifies HGF as a critical mediator of synovial inflammation in RA. The mechanistic insights provided here pave the way for the development of innovative strategies targeting the HGF-c-Met axis, potentially addressing unmet needs in the management of refractory RA.

## Data Availability

The datasets presented in this study can be found in online repositories. The names of the repository/repositories and accession number(s) can be found in the article/**Supplementary Material**.
