# Supplementary material for: Hepatocyte growth factor as a driver of synovial inflammation and therapeutic resistance in rheumatoid arthritis
Source: Front Immunol. 2026 Jan 30;17:1718591. doi: 10.3389/fimmu.2026.1718591 (PMC12901490; doi:10.3389/fimmu.2026.1718591)
Supplement: Supplementary file 1 [file Table1.docx]

**Supplementary Table 1. Clinical characteristics of 66 patients with rheumatoid arthritis**

| **Variable** | **RA patients (N = 66)** |
| --- | --- |
| Age at enrollment | 67.5 [59–73] |
| Disease duration (months) | 105.5 [72.3–183] |
| Sex (Female) | 50 (75.8%) |
| RF (IU/mL) | 31.7 [11.1–75.9] |
| RF-positive (>15 IU/mL) | 47 (71.2%) |
| Anti-CCP antibody, U/mL | 21.1 [0.6–174] |
| Anti-CCP antibody-positive (>4.5 U/mL) | 46 (69.7%) |
| CRP (mg/dL) | 0.1 [0.1–0.1] |
| ESR (mm/h) | 22.5 [11.0–40.5] |
| Methotrexate use, n (%) | 47 (71.2%) |
| Methotrexate dose (mg/week) | 8.0 [4.0–10.0] |
| Prednisolone use, n (%) | 19 (28.8%) |
| Prednisolone dose (mg/day) | 5.0 [2.0–5.0] |
| bDMARDs use, n (%) | 36 (54.5%, TNFi 21, aIL-6R 8, CTLA4-Ig 7) |
| tsDMARDs use, n (%) | 1 (1.5%) |
| DAS28-ESR | 2.7 [2.0–3.4] |
| DAS28-CRP | 1.6 [1.3–2.3] |
| SDAI | 2.2 [0.7–5.9] |
| CDAI | 1.9 [0.6–5.1] |

Results are expressed as median [interquartile range] for continuous variables, or the number (%) for nominal variables.

Anti-CCP antibody, anti-cyclic citrullinated peptide antibody; aIL-6R, anti-interleukin-6 receptor antibodies; bDMARDs, biologic disease modifying-antirheumatic drugs; CDAI, Clinical Disease Activity Index; CRP, C-reactive protein; CTLA4-Ig, cytotoxic T lymphocyte-associated antigen-4-Ig; DAS28-CRP, 28-joint disease activity score using C-reactive protein; DAS28-ESR, 28-joint disease activity score using erythrocyte sedimentation rate; ESR, erythrocyte sedimentation rate; RF, rheumatoid factor; SDAI, simplified Disease Activity Index; TNFi, tumour necrosis factor inhibitor; tsDMARDs, targeted synthetic disease modifying-antirheumatic drugs.

**Supplementary Table ２. Comparison of plasma protein levels between healthy controls and patients with RA.**

| Parameters | HC | RA | *p* |
| --- | --- | --- | --- |
| IL-1β | 3.42 [2.24-5.07] | 4.72 [3.42-6.10] | 0.081 |
| IL-2 | 6.93 [6.93-10.98] | 9.57 [7.40-13.18] | 0.18 |
| IL-4 | 37.17 [26.82-53.60] | 54.68 [41.69-75.12] | 0.062 |
| IL-6 | 2.01 [1.94-3.89] | 4.03 [2.82-6.87] | **0.014** |
| IL-10 | 1.77 [1.77-2.25] | 2.65 [1.77-3.78] | 0.051 |
| IL-17 | 3.26 [3.26-3.36] | 3.26 [3.26-3.65] | 0.50 |
| IFN-α | 2.69 [2.69-2.71] | 2.69 [2.69-2.75] | 0.45 |
| IFN-β | 12.37 [12.37-12.37] | 12.37 [12.37-12.37] | 1 |
| IFN-γ | 8.40 [6.92-10.11] | 11.32 [7.13-18.36] | 0.065 |
| TNF-α | 2.33 [1.95-4.87] | 6.77 [4.27-9.80] | **0.0046** |
| Dkk-1 | 262.4 [199.7-364.9] | 328.2 [228.8-473.1] | 0.27 |
| CCL2 | 224.4 [171.8-288.3] | 250.2 [196.3-318.2] | 0.50 |
| CXCL10 | 29.23 [16.83-41.75] | 45.69 [34.23-83.53] | **0.018** |
| HGF | 23.92 [11.37-32.55] | 51.14 [37.35-67.14] | **0.0003** |

Data are shown as median [interquartile range] and Mann-Whitney U test was used. CCL2, C-C motif chemokine ligand 2; CXCL10, C-X-C motif chemokine ligand 10; Dkk-1, dickkopf-1; HC, healthy controls; HGF, hepatocyte growth factor; IFN, interferon; IL, interleukin; RA, rheumatoid arthritis; TNF, tumour necrosis factor.
