# Supplementary material for: Hepatocyte growth factor as a driver of synovial inflammation and therapeutic resistance in rheumatoid arthritis
Source: Front Immunol. 2026 Jan 30;17:1718591. doi: 10.3389/fimmu.2026.1718591 (PMC12901490; doi:10.3389/fimmu.2026.1718591)
Supplement: Supplementary file 3 [file Table3.docx]

Supplementary Methods

**Quantitative PCR**

RNA-Quick Purification Kit (QIAGEN , #74134, Hilden, GERMANY) was used to isolate the total RNA. Complementary DNA (cDNA) was synthesized by the ReverTraAce kit (#FSQ-201; Toyobo, Osaka, Japan). qPCR was performed through the PowerTrack SYBR Green Master Mix (#A46109, Thermo Fisher Scientific, USA). Primer sequences are as follows: HGF forward primer: : GCT ATC GGG GTA AAG ACC TAC A; HGF reverse primer: CGT AGC GTA CCT CTG GAT TGC ; c-MET forward: AGC AAT GGG GAG TGT AAA GAG G; c-MET reverse: CCC AGT CTT GTA CTC AGC AAC and IL-6 forward primer: : AGC CAC TCA CCT CTT CAG AAC GAA ; IL-6 reverse primer: AGT GCC TCT TTG CTG CTT TCA CAC.

**Immunofluorescence staining of human synovial tissues**

Freshly isolated synovial tissues were embedded in an optical cutting temperature (OCT) compound (Cat No.4583, Sakura, Finetek, USA) before being frozen in liquid nitrogen. Cryosections (5-μm-thick) were cut using a Leica CM1950 machine and mounted on 2.0-μm-thick PEN-Membrane slides (#MAS-04, Matsunami, Japan). They were fixed in 4% formalin buffer at 4°C for 30 minutes. The tissue sections were washed three times with PBS-T and then blocked with 5% bovine serum albumin (BSA) in PBS-T for 1 hour. Subsequently, the samples were incubated overnight at 4°C with rabbit anti-HGF antibody (1:100, #ab313408, Abcam) or rabbit anti-c-Met antibody (1:100, Abcam, #ab51067) and mouse anti-CD90 antibody (1:100, #ab181469, Abcam) mixed in Signal Enhancer HIKARI solution (#L4G6882, Nacalai Tesque, Kyoto, Japan). After three washes with PBST, the tissue sections were incubated with corresponding secondary antibodies Alexa Fluor 488-conjugated goat anti-rabbit antibody (1:200, #A11034, Invitrogen) and Alexa Fluor 594-conjugated goat anti-mouse antibody (1:200, #A11005, Invitrogen) at room temperature (RT) for 1 hour. For nuclear staining, 4′,6-diamidino-2-phenylindole (DAPI) (1/500, #340-07971, Doijndo) was used at RT for 20 minutes. Finally, the tissue sections were examined using BZ-X700 All-in-One fluorescence microscope (KEYENCE, Osaka, Japan).

**Immunofluorescence staining of mouse synovial tissues**

The isolation and fixation of mouse synovial tissues were conducted using the same protocol as for human synovial tissues. Immunostaining was performed using the following primary antibodies: mouse anti-HGF antibody (1:100, #MA514160, Invitrogen) and rabbit anti-CD90 antibody (1:100, #ab307736, Abcam). The corresponding secondary antibodies were Alexa Fluor 488-conjugated goat anti-rabbit antibody (1:200, #A11034, Invitrogen) and Alexa Fluor 594-conjugated goat anti-mouse antibody (1:200, #A11005, Invitrogen).

**Synovial fibroblast migration assay**

For the wound-healing assay, confluent synovial fibroblasts (SFs) derived from three distinct rheumatoid arthritis (RA) patient lines were cultured in 12-well plates. A uniform, cell-free wound area was generated in each well by scratching the monolayer with a sterile pipette tip. The cells were then incubated in DMEM containing 10% FCS, supplemented with either the vehicle control (DMSO) or hepatocyte growth factor (50 ng/mL). Images were captured using a confocal microscope at 10× magnification at 0, 8, and 16 hours after wounding. Cell migration was quantified by measuring the width of the wound in each well. The wound area at 0 hours was defined as 100% open, and the percentage of wound closure at subsequent time points was calculated relative to this baseline (1).

**Isolation and cell culture of human CD14^+^** **monocytes**

Peripheral blood mononuclear cells (PBMC) were isolated from whole blood by density gradient centrifugation (Lymphoprep; Stemcell Technologies, Canada). A magnetic sorting of CD14^+^ cells was performed by adding MACS superparamagnetic microbeads (Cat No.00108, Miltenyi‐Biotec, Germany) conjugated with monoclonal anti‐human CD14 antibodies (Cat No.0009, Miltenyi‐Biotec), according to the manufacturer’s instructions. Freshly isolated CD14^+^ monocytes (≥95%) were cultured in RPMI 1640 (Thermo Fisher Scientific) supplemented with 10% FCS, HEPES (20 mM), penicillin–streptomycin.

**Immunofluorescence staining of human CD14+ monocytes**

Human CD14+ monocytes were plated at a density of 20,000 cells per well in 8-well Labtek chamber slides. After 3 days, the cells were divided into 4 groups. The first group served as the control, while the second, third, and fourth groups were treated with LTA-SA (1 μg/mL, # tlrl-pslta, Invivogen), FLA-ST (100 ng/mL, # tlrl-stfla, Invivogen), and LPS-PG (20 ng/mL,# tlrl-pglps, Invivogen), respectively for 6 hours. After treatment, the cells were washed with PBS and fixed with 4% paraformaldehyde in PBS for 30 minutes, and then blocked with 10% BSA in PBS for 1 hour at RT. Subsequent staining procedure was same as fibroblasts.

**Analysis of single cell RNA sequencing data from the Accelerating Medicines Partnership (AMP) projects**

To evaluate the expression of HGF and c-Met in each cell type in synovium tissue from patients with RA, we analyzed publicly available single-cell data from the AMP project (2). The R package Seurat (ver 4.4.0) (3) was used for data processing. Briefly, cells with outlier values for the number of genes, total RNA count, or percentage of mitochondrial gene expression were excluded, followed by log-normalization, scaling, dimension reduction, and Uniform Manifold Approximation and Projection (UMAP) visualization. Cell-type annotation was performed using canonical markers (4, 5). All processes are documented in the provided GitHub repository: <https://github.com/takeshiiwasaki/HGF>.

**Arthritis score in SKG mouse**

Arthritis was induced by a single intraperitoneal injection of 2 mg of Zymosan (Z4250, Merck, Japan) on day 0 (6). Joint swelling was assessed by visual inspection and scored as follows: 0, no swelling; 0.1, swelling of one toe joint; 0.5, mild ankle swelling; and 1.0, severe ankle swelling. Scores for all toes and ankles were summed for each mouse (6).

**References**

1. Choi C, Jeong W, Ghang B, Park Y, Hyun C, Cho M, et al. Cyr61 synthesis is induced by interleukin-6 and promotes migration and invasion of fibroblast-like synoviocytes in rheumatoid arthritis. Arthritis Res Ther. 2020;22(1):275.

2. Zhang F, Wei K, Slowikowski K, Fonseka CY, Rao DA, Kelly S, et al. Defining inflammatory cell states in rheumatoid arthritis joint synovial tissues by integrating single-cell transcriptomics and mass cytometry. Nat Immunol. 2019;20(7):928-42.

3. Hao Y, Hao S, Andersen-Nissen E, Mauck WM, 3rd, Zheng S, Butler A, et al. Integrated analysis of multimodal single-cell data. Cell. 2021;184(13):3573-87 e29.

4. Wilk AJ, Rustagi A, Zhao NQ, Roque J, Martinez-Colon GJ, McKechnie JL, et al. A single-cell atlas of the peripheral immune response in patients with severe COVID-19. Nat Med. 2020;26(7):1070-6.

5. Xu C, Prete M, Webb S, Jardine L, Stewart BJ, Hoo R, et al. Automatic cell-type harmonization and integration across Human Cell Atlas datasets. Cell. 2023;186(26):5876-91 e20.

6. Yoshitomi H, Sakaguchi N, Kobayashi K, Brown GD, Tagami T, Sakihama T, et al. A role for fungal beta-glucans and their receptor Dectin-1 in the induction of autoimmune arthritis in genetically susceptible mice. J Exp Med. 2005;201(6):949-60.
