# Supplementary material for: Hepatocyte growth factor as a driver of synovial inflammation and therapeutic resistance in rheumatoid arthritis
Source: Front Immunol. 2026 Jan 30;17:1718591. doi: 10.3389/fimmu.2026.1718591 (PMC12901490; doi:10.3389/fimmu.2026.1718591)
Supplement: Supplementary file 4 [file Table4.docx]

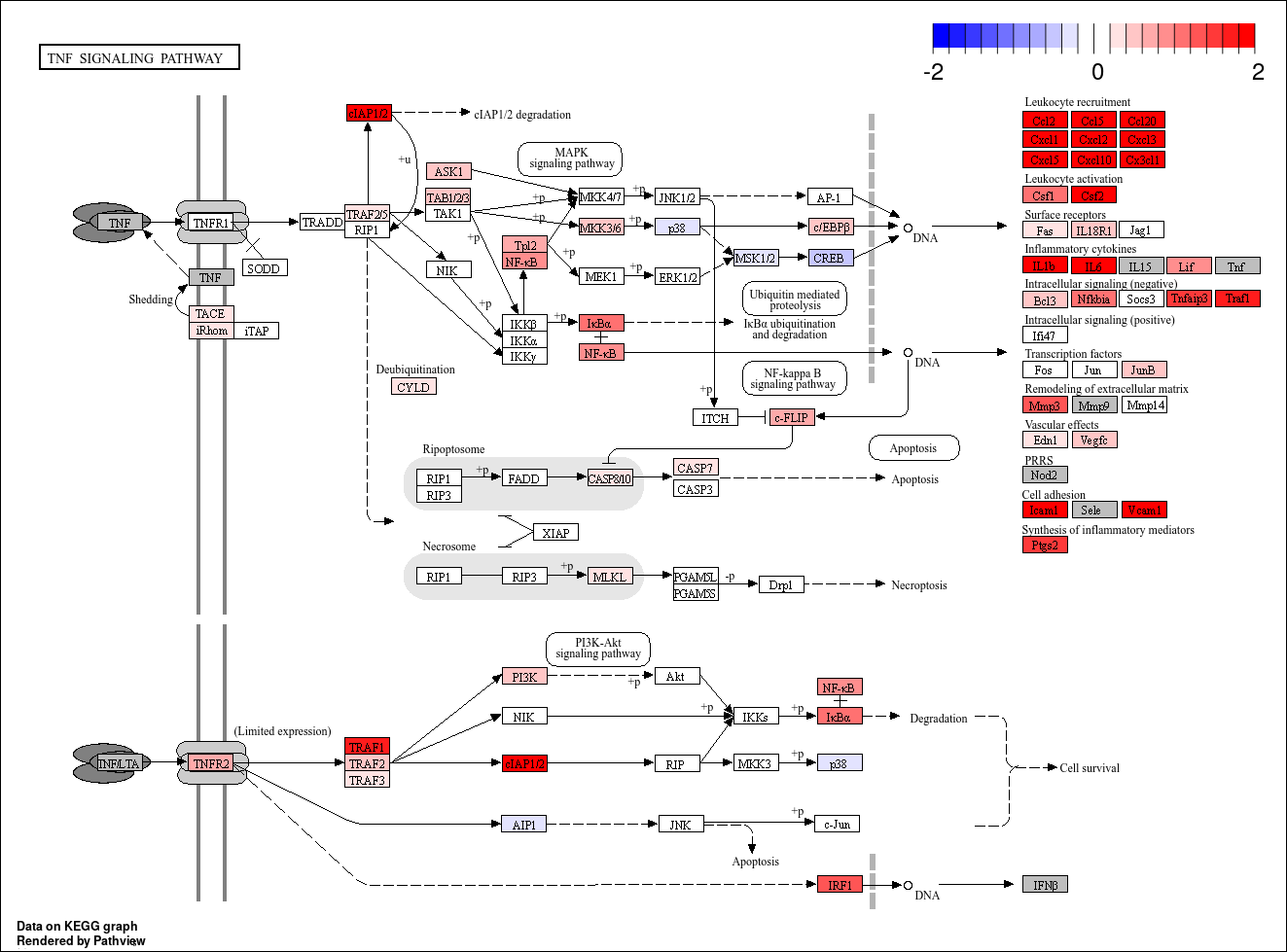


**Supplementary Figure 1.** **Mapping of differentially expressed genes on the TNF signaling pathway using Pathview.** Log2 (fold change) values following hepatocyte growth factor treatment on the corresponding transcripts are shown in color. Gray indicates genes for which differential expression test results were not available.


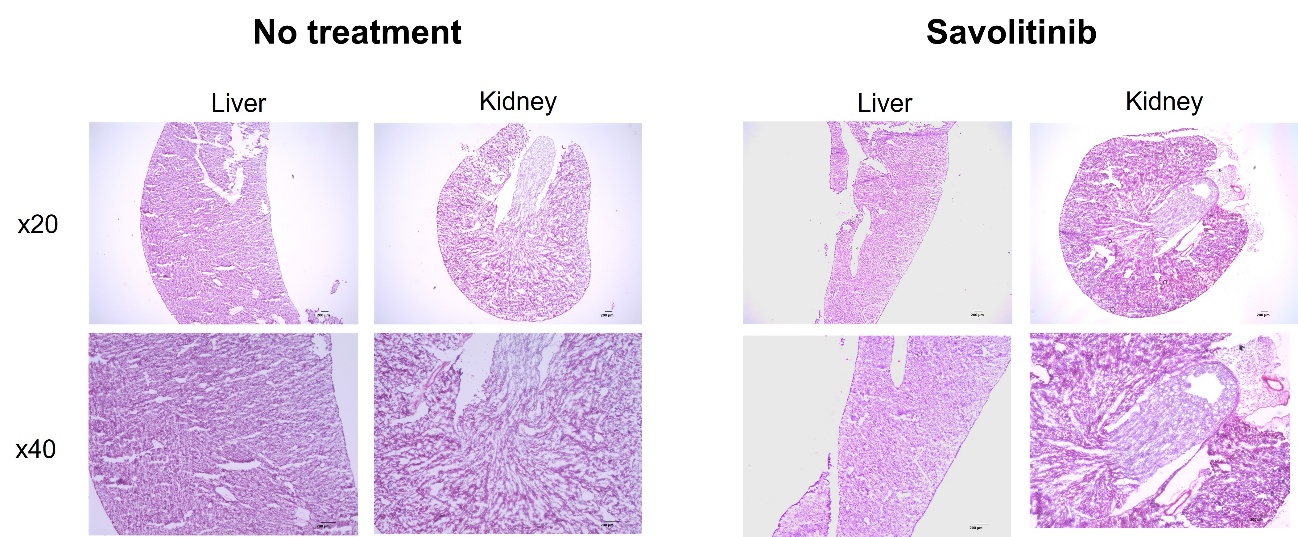


**Supplementary Figure 2.** **No apparent abnormalities in the liver or kidneys of savolitinib-treated mice.** Histological evaluation using hematoxylin-eosin (H&E) staining revealed no apparent abnormalities in the liver or kidneys of untreated and savolitinib-treated mice. Representative images from 4 mice are shown. Scale bars indicate 200 μm.


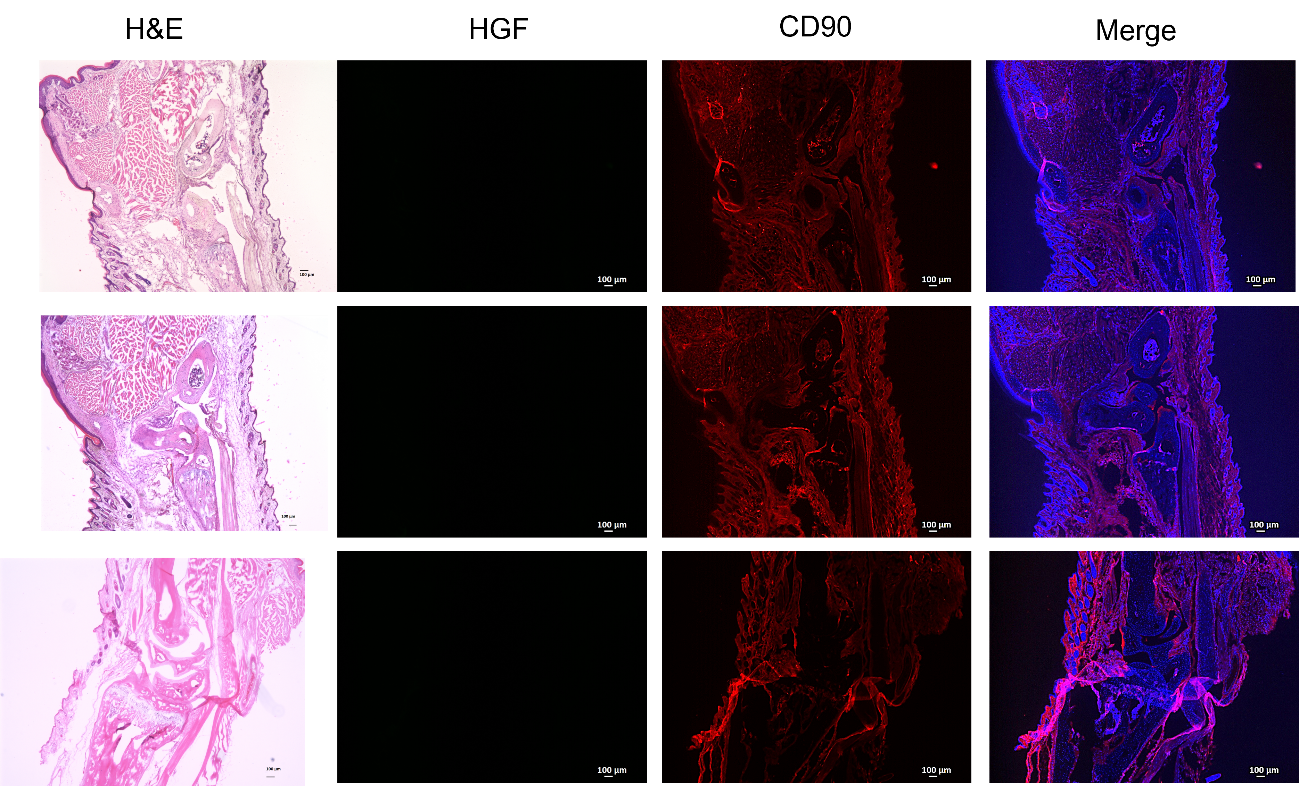


**Supplementary Figure 3.** **Baseline expression of HGF in mice without zymosan-induced arthritis.** Hepatocyte growth factor (HGF) expression was evaluated in mice that did not receive zymosan and therefore did not develop arthritis (Figure 5A). Representative hematoxylin-eosin (H&E) staining and immunostaining (HGF and CD90) of joints from 3 mice are shown. Scale bars indicate 100 μm.
